# Fluid overload is associated with increased 90-day mortality in AML patients undergoing induction chemotherapy

**DOI:** 10.1007/s00277-021-04593-x

**Published:** 2021-07-25

**Authors:** Olivier Ballo, Fagr Eladly, Sebastian Koschade, Stefan Büttner, Jan Alexander Stratmann, Uta Brunnberg, Eva-Maria Kreisel, Franziska Frank, Sebastian Wagner, Björn Steffen, Hubert Serve, Fabian Finkelmeier, Christian H. Brandts

**Affiliations:** 1 Department of Medicine, Hematology/Oncology, University Hospital, Goethe University, Frankfurt, Germany; 2 Department of Medicine, Nephrology, University Hospital, Goethe University, Frankfurt, Germany; 3 German Cancer Consortium (DKTK) and German Cancer Research Center (DKFZ), Heidelberg, Germany; 4 Department of Medicine, Gastroenterology, Hepatology and Endocrinology, University Hospital, Goethe University, Frankfurt, Germany; 5 University Cancer Center Frankfurt (UCT), University Hospital, Goethe University, Frankfurt, Germany

**Keywords:** Fluid overload, Acute myeloid leukemia, Induction chemotherapy, Survival, Intensive care treatment

## Abstract

Treatment‐related complications contribute substantially to morbidity and mortality in acute myeloid leukemia (AML) patients undergoing induction chemotherapy. Although AML patients are susceptible to fluid overload (FO) (e.g., in the context of chemotherapy protocols, during sepsis treatment or to prevent tumor lysis syndrome), little attention has been paid to its role in AML patients undergoing induction chemotherapy. AML patients receiving induction chemotherapy between 2014 and 2019 were included in this study. FO was defined as ≥5% weight gain on day 7 of induction chemotherapy compared to baseline weight determined on the day of admission. We found FO in 23 (12%) of 187 AML patients undergoing induction chemotherapy. Application of >100 ml crystalloid fluids/kg body weight until day 7 of induction chemotherapy was identified as an independent risk factor for FO. AML patients with FO suffered from a significantly increased 90-day mortality rate and FO was demonstrated as an independent risk factor for 90-day mortality. Our data suggests an individualized, weight-adjusted calculation of crystalloid fluids in order to prevent FO-related morbidity and mortality in AML patients during induction chemotherapy. Prospective trials are required to determine the adequate fluid management in this patient population.

## Introduction

Acute myeloid leukemia (AML) is a hematological malignancy arising from a clonal proliferation of myeloid precursors losing their ability to differentiate into mature functional blood cells. A curative therapy approach can only be achieved by intensive induction chemotherapy. During the past 30 years, advances in intensified chemotherapy, allogenic stem cell transplantation (SCT), and supportive care resulted in increased survival rates (1–4). Nevertheless, treatment‐related complications contribute to a substantial proportion of morbidity and a mortality rate of 8–15% (5, 6).

In critically ill patients, fluid overload (FO) is associated with a higher risk of kidney failure, increased mortality, and the risk of irreversible organ damage, especially pulmonary dysfunction due to capillary leakage (7–9). Furthermore, in critically ill patients, endothelial damage due to inflammatory cytokine milieu may exacerbate FO (10, 11). Although AML patients are prone to positive fluid balance due to excessive fluid input (e.g., in the context of chemotherapy protocols, during sepsis treatment or due to blood transfusions), very little attention has been paid to its role in AML patients undergoing induction chemotherapy. Chamoun et al. have shown that 63 (34%) of 187 patients with acute promyelocytic leukemia (APL) develop FO during induction treatment. FO was associated with treatment on intensive care unit (ICU) and endotracheal intubation. Increased LDH and creatinine levels, low albumin, and the total volume of blood product transfusions were identified as risk factors for the development of FO during induction therapy (12). Miller et al. focused on pediatric AML patients; FO occurred in 30 (28.6%) of 105 pediatric AML patients during induction chemotherapy. FO was more likely in patients aged ≥ 10 years and also in patients with infection, hyperleukocytosis, and low hemoglobin levels at diagnosis (13). Potentially modifiable risk factors such as the volume of crystalloid fluids applied during induction were not analyzed. In the context of allogenic SCT, FO was correlated with a higher non-relapse mortality and impaired overall survival (OS) (14, 15).

To our knowledge, there is no study investigating the role of FO in patients with AML undergoing induction chemotherapy. The objectives of this study are to determine the incidence of FO in AML patients, to identify associated risk factors, and to evaluate its impact on the clinical course during induction chemotherapy.

## Materials and methods

### Study design and treatment protocols

In this single-center study, we retrospectively included all patients aged ≥18 with AML (excluding acute promyelocytic leukemia) who underwent intensive induction chemotherapy between 2014 and 2019. There is no standard definition for FO in hematological patients (12–14). Since AML patients suffer from body weight loss especially during initial diagnosis leading to possible underestimation FO incidence, we chose a comparably low cutoff at 5%. As the aim of this study was to evaluate the possible adverse impact of non-individualized administration of crystalloid fluids recommended as concomitant medication by current treatment protocols, we chose day 7 of induction chemotherapy to screen for FO. Hence, FO was defined as ≥5% weight gain on day 7 of induction chemotherapy with respect to a baseline weight determined on the day of admission. Patient body weight was measured on daily basis. All patients received echocardiographic assessment before initiation of treatment to exclude congestive heart failure. Data sources for determining causes of death and/or reasons for ICU transfer include documentation within the electronic patient record and the ICU letter written at discharge or death. Standard induction chemotherapy was the so-called *7+3* regime; cytarabine 100 mg/m^2^ administered intravenously (IV) continuously for 7 days is combined with daunorubicin 60 mg/m^2^ applied as a 30-min IV infusion on days 3, 4, and 5 (16). As recommended by national treatment protocols in general, 1000ml/day of crystalloid fluids was applied daily on all 7 days of induction chemotherapy (17). Patients under the age of 60 received a second induction therapy with *7+3* if early blast clearance was achieved in d15 bone marrow blood evaluation or with *HAM protocol* (cytarabine 3000mg/m^2^ was administered by 3-h IV infusion every 12 h on days 1 through 3 and mitoxantrone 10 mg/m^2^ by 30-min IV infusion on days 3, 4, and 5) if blast clearance was not achieved on d15 bone marrow blood evaluation (18). Patients above the age of 60 received only a second induction chemotherapy with HAM (with reduced cytarabine dose of 1000 mg/m^2^), if the first induction therapy cycle was not sufficient to achieve bone marrow blast clearance on d15 (19). In case of a complete remission (CR) after induction chemotherapy with 7+3 alone or with 7+3 and HAM, patients went on to receive a consolidation treatment with either high-dose cytarabine or an allogenic SCT. Response assessment was performed in accordance with the European Leukemia Net (ELN) recommendations from 2010 (20). Patients routinely received routine antimicrobial prophylaxis with levofloxacin and posaconazole daily as recommended by current guidelines (21, 22). A day with fever was defined as a body temperature increase above ≥38.3°C once or ≥38.0 °C on two consecutive days (23). If fever or a significant increase of C-reactive protein (CRP) was found, antibiotic prophylaxis was replaced by intravenous broad-spectrum antibiotics. Blood testing (hematology, liver and kidney function, coagulation, inflammation markers) was performed every other day routinely. Until August of 2015, AML patients undergoing intensive induction chemotherapy received red blood cell (RBC) concentrates if Hb levels dropped below 8 g/dl. In September 2015, the institutional transfusion guideline was changed and AML patients received RBC concentrates if Hb levels dropped below 7 g/dl. In case of anemia symptoms, earlier RBC transfusion was allowed if deemed clinically indicated. Platelet concentrates were given if platelet count dropped below 10/nl or earlier if the patient presented with hemorrhage (24).

The study was performed in accordance with the 2013 Helsinki declaration. Patients provided informed written consent to retrospective data extraction from patient charts and patient data was provided after approval by the local ethics committee. The ethics committee waived the requirement for informed consent for deceased patients. In addition, the majority of patients were also enrolled in the AML registry of the Study Alliance Leukemia. After ethics approval, patient data was retrieved from the clinical cancer registry of the University Cancer Center (UCT) Frankfurt, complemented by data directly from the medical records and fully anonymized. Data analysis was performed on anonymized data.

### Statistical analysis

This study was designed as a retrospective cohort study. Patients were followed until death or last contact. Dates of treatment start and finish with induction chemotherapy were assessed separately. Continuous variables are shown as median ± range and categorical variables are reported as frequencies and percentages. All continuous variables were tested for normality and were analyzed by using Student’s *t*-test or the Wilcoxon-Mann-Whitney test accordingly. A chi-squared test was used for binary variables. Death rates were analyzed by the Kaplan-Meier method and curves were compared by log-rank test. Predictors of survival were determined using a univariate Cox regression hazard model. Death was recorded as an event. Risk factors for FO were assessed by uni- and multivariate logistic regression with variable elimination of non-significant predictors by forward selection. Statistical analysis was performed with SPSS (Version 27.0, IBM, Armonk, NY).

## Results

### Baseline characteristics of AML patients with and without FO

A total of 187 patients diagnosed with AML between 2014 and 2019 that underwent intensive induction therapy were included in this retrospective analysis. According to our definition, 23 (12.3%) AML patients suffered from FO on day 7 of induction chemotherapy and 164 (87.7%) AML patients did not have FO on day 7 of induction chemotherapy (Table 1). Median age was 60 years (range 23–73) in AML patients with FO and 58 years (range 18–85) in AML patients without FO (*p*=0.321). Ten (43.5%) AML patients with FO had female sex compared to 58 (28.1%) AML patients without FO (*p*=0.820). AML patients with FO had a significantly lower body weight on the day of admission than AML patients without FO (73.1 kg, range 48.9–115.3 vs. 81.2 kg, range 40–140.7, *p*=0.015). The percentage of patients with abnormalities in left ventricular ejection fraction or diastolic relaxation was similar in AML patients with and without FO (4.3% vs. 2.4%, *p*=0.485 and 34.8% vs. 42.1%, *p*=0.652 respectively). There was no significant difference between the two cohorts with respect to the WHO classifications and AML risk groups according to the European Leukemia Net (ELN) recommendations from 2010 were equally distributed (*p*=0.588 and 0.915 respectively) (20). Blood counts as well as albumin and C-reactive protein (CRP) levels were similar in AML patients with and without FO. Median creatinine levels were 0.89 mg/dl (0.51–1.61) in AML patients with FO and 0.87 mg/dl (0.45–2.21) in AML patients without FO (*p*=0.767).

**Table 1:** Baseline characteristics of AML patients with and without fluid overload

**Characteristic**	**All**	**AML patients with FO**	**AML patients without FO**	***p*-value**
**Number of patients (*n*, %)**	187 (100)	23 (12.3)	164 (87.7)	
**Median age (median, range)**	58 (18–85)	60 (23–73)	58 (18–85)	0.321
**Female sex (*n*, %)**	56 (29.95)	10 (43.5)	46 (28.1)	0.820
Body weight kg (median, range)*	80.6 (40–140.7)	73.1 (48.9–115.3)	81.2 (40–140.7)	**0.015**
**Normal left ventricular ejection fraction (*n*, %)**	182 (97.3)	22 (95.7)	160 (97.6)	0.485
**Mildly abnormal left ventricular ejection fraction (*n*, %)**	5 (2.7)	1 (4.3)	4 (2.4)	0.485
**Diastolic relaxation abnormality (*n*, %)**	77 (41.2)	8 (34.8)	69 (42.1)	0.652
**AML with recurrent genetic abnormalities (*n*, %)**	72 (38.5)	8 (34.8)	64 (39.0)	0.588
**AML with myelodysplasia-related changes (*n*, %)**	49 (26.2)	6 (26.1)	43 (26.2)	0.588
**Therapy-related myeloid neoplasms (*n*, %)**	5 (2.7)	3 (13.0)	2 (1.2)	0.588
**AML not otherwise specified (*n*, %)**	59 (31.6)	7 (30.4)	52 (31.7)	0.588
**Favorable ELN risk group (*n*, %)**	39 (20.9)	4 (17.4)	35 (21.3)	0.915
**Intermediate-I ELN risk group (*n*, %)**	68 (36.4)	10 (43.5)	58 (18.9)	0.915
**Intermediate-II ELN risk group (*n*, %)**	35 (18.7)	4 (17.4)	31 (18.9)	0.915
**Adverse ELN risk group (*n*, %)**	41 (21.9)	5 (21.7)	36 (22.0)	0.915
Albumin g/dl (median, range)*	3.9 (2–5.2)	3.7 (2.1–5)	3.9 (2–5.2)	0.376
White blood count/nl (median, range)*	7.72 (0.38–324.73)	11.54 (0.60–232.49)	6.75 (0.38–324.73)	0.245
Hemoglobin g/dl (median, range)*	9.0 (3.5–15.1)	9 (5–14.4)	9.1 (3.5–15.1)	0.632
Platelet count/nl (median, range)*	64 (3–836)	82 (10–283)	64 (3–836)	0.627
Creatinine mg/dl (median, range)*	0.88 (0.45–2.21)	0.89 (0.51–1.61)	0.87 (0.45–2.21)	0.767
C-reactive protein mg/dl (median, range)*	1.98 (0.01–34.66)	3.93 (0.04–19.80)	1.76 (0.01–34.66)	0.084

*At admission. All *p*-values reported are two-sided. Statistical significance was defined as *p*≤0.05, significant p-values are presented in bold

### Clinical and laboratory findings in AML patients with and without FO

Three (13.0%) AML patients with FO and 13 (8.6%) AML patients without FO received leukapheresis for initial treatment of hyperleukocytosis (*p*=0.411). Median days from admission to day 7 of induction chemotherapy was 10 (7–19) in AML patients with FO and 9 (7–35) in AML patients without FO (*p*=0.978). In that period of time, AML patients with FO received a median of 8000 ml (0–22000) of crystalloid fluids compared to 7000 ml (0–28000) in AML patients without FO (*p*=0.527) (Table 2). Sixteen (69.6%) AML patients with FO received more than 100 ml per kg body weight until day 7 of induction chemotherapy, significantly more when compared to 47 (28.7%) AML patients without FO (*p*=0.043). Serum albumin levels measured during the hospital stay for induction chemotherapy of AML patients with FO were similar to those of AML patients without FO (3.3 g/dl, range 2.1–3.8 vs. 3.4 g/dl, range 2.25–4.2, *p*=0.117). Median serum creatinine levels were 0.7 mg/dl (0.47–1.38) in AML patients with FO and 0.76 mg/dl (0.38–3.03) in AML patients without FO (*p*=0.417). Median C-reactive protein (CRP) levels were more than twice as high in AML patients with FO as in AML without FO (6.5 mg/dl, range 2.0–24.2 vs. 2.9 mg/dl, range 0.3–16.0, *p*<0.001). Hemoglobin and platelet counts were 8.2 g/dl (7.5–10) and 23.5/nl (7–51) in AML patients with FO and 8.3 g/dl (6.4–13.2) and 27.5/nl (5.5–210) in AML patients without FO (*p*=0.784 and 0.170 respectively). The median number of platelet concentrates transfused until day 7 of induction chemotherapy was 2 (0–8) in AML patients with FO and 1 (0–18) in AML patients without FO (*p*=0.067) and the number of RBC concentrates was 4 (0–10) and 2 (0–16) respectively (*p*=0.044). 56.5% of AML patients with FO received diuretics until day 7 of induction chemotherapy compared to 20.1% in AML patients without FO (*p*<0.001). Body weight on day 7 of induction chemotherapy was 79.9 kg (52.1–124.8) in AML patients with FO and 81.3 kg (49–145.2) in AML patients without FO (*p*=0.448). AML patients with FO had 6 (0–24) days with fever during the hospital stay for induction chemotherapy and 5 (21.7%) required treatment on ICU compared to 5 (0–31) days with fever and 23 (14%) required treatment on ICU in AML patients without FO (*p*=0.377 and 0.332 respectively). The length of the hospital stay did not differ significantly between both cohorts (46 days, 15–70 in AML patients with FO vs. 49 days, 16–127 in AML patients without FO, *p*=0.470). Response rates in terms of day 15 bone marrow blast clearance and complete remission (CR) rates after induction chemotherapy were not different in AML patients with and without FO (*p*=0.280 and 0.585 respectively). Eight (34.8%) AML patients with FO received allogenic SCT as consolidation therapy compared to 58 (21.3%) AML patients without FO (*p*=1.000). AML patients with FO had a higher 90-day mortality (*n*=4, 17.4% vs. 6, 3.7 %, *p*=0.022). Overall mortality did not differ significantly (*n*=11, 47.8% vs. 55, 33.5%, 0.179).

**Table 2:** Clinical findings in AML patients with and without fluid overload

**Characteristic**	**AML patients with FO**	**AML without FO**	***p*-value**
**Number of patients (*n*, %)**	23 (12.3)	164 (87.7)	
**Leukapheresis for initial treatment of hyperleukocytosis (*n*, %)**	3 (13.0)	13 (8.6)	0.411
**Days from admission to day 7 of induction chemotherapy (median, range)**	10 (7–19)	9 (7–35)	0.978
Crystalloid fluids applied in ml (median, range)*	8000 (0–22000)	7000 (0–28000)	0.527
Patients receiving >100 ml crystalloid fluids/kg bodyweight*	16 (69.6)	47 (28.7)	**0.043**
**Albumin g/dl (median, range)**^**O**^	3.3 (2.1–3.8)	3.4 (2.25–4.2)	0.117
**Creatinine mg/dl (median, range)**^**O**^	0.70 (0.47–1.38)	0.76 (0.38–3.03)	0.417
**C-reactive protein mg/dl (median, range)**^**O**^	6.5 (2.0–24.2)	2.9 (0.3–16.0)	**<0.001**
**Hemoglobin g/dl (median, range)**^**O**^	8.2 (7.5–10.0)	8.3 (6.4–13.2)	0.784
**Platelet count/nl (median, range)**^**O**^	23.5 (7–51)	27.5 (5.5–210)	0.170
Transfused platelet concentrates (median, range)*	2 (0–8)	1 (0–18)	0.067
Transfused red blood cell concentrates (median, range)*	4 (0–10)	2 (0–16)	**0.044**
Patients receiving diuretics (*n*, %)*	13 (56.5)	33 (20.1)	**<0.001**
**Body weight on day 7 (median, range)**	79.9 (52.1–124.8)	81.3 (49–145.2)	0.448
**Days with fever (median, range)**	6 (0–24)	5 (0–31)	0.377
**Patients requiring treatment on intensive care unit (*n*, %)**	5 (21.7)	23 (14.0)	0.332
**Length of hospital stay (median, range)**	46 (15–70)	49 (16–127)	0.470
**Day 15 bone marrow blast clearance (*n*, %)**	10 (43.5)	78 (47.6)	0.280
**Complete remission after induction chemotherapy (*n*, %)**	13 (56.5)	120 (73.2)	0.585
**Stem cell transplantation as consolidation therapy (*n*, %)**	8 (34.8)	58 (21.3)	1.000
**90-day mortality (*n*, %)**	4 (17.4)	6 (3.7)	**0.022**
**Overall mortality (*n*, %)**	11 (47.8)	55 (33.5)	0.179

*From day of admission until day 7 of induction chemotherapy. ^**O**^Median values of the hospital stay for induction chemotherapy. All *p*-values reported are two-sided. Statistical significance was defined as *p*≤0.05, significant p-values are presented in bold

### Risk factors for FO in AML patients undergoing induction chemotherapy

To further analyze risk factors for FO in AML patients undergoing induction chemotherapy, a uni- and multivariate logistic regression model was performed. The nominal dichotome variables included in this model were as follows: age >60 years, treatment on ICU, fever days >5, patients receiving >100 ml crystalloid fluids/kg body weight until day 7 of induction chemotherapy, and CRP levels >10 mg/dl. As shown in Table 3, patients receiving >100 ml crystalloid fluids/kg body weight until day 7 of induction chemotherapy was an independent risk factor for FO in AML patients undergoing induction chemotherapy.

**Table 3:** Logistic regression analysis of risk factors for fluid overload

Parameter	OR	95% CI	*P* value	OR	95 % CI	*P* value
	Univariate analysis			Multivariate analysis		
**Age >60 years**	1.746	0.724–4.211	0.215			
**Treatment on intensive care unit**	1.703	0.576–5.037	0.336			
**Fever days >5**	1.659	0.667–4.128	0.276			
Patients receiving >100 ml crystalloid fluids/kg body weight*	2.780	1.086–7.116	**0.033**	2.780	1.086–7.116	**0.033**
**CRP level > 10 mg/dl**	2.976	0.959–9.233	0.059			

*Including crystalloid fluids from day of admission until day 7 of induction chemotherapy. *CI*, confidence interval; *OR*, odds ratio. All *p*-values reported are two-sided. Statistical significance was defined as *p*≤0.05, significant p-values are presented in bold

### Risk factors for 90-day mortality in AML patients undergoing induction chemotherapy

**Fig. 1:**
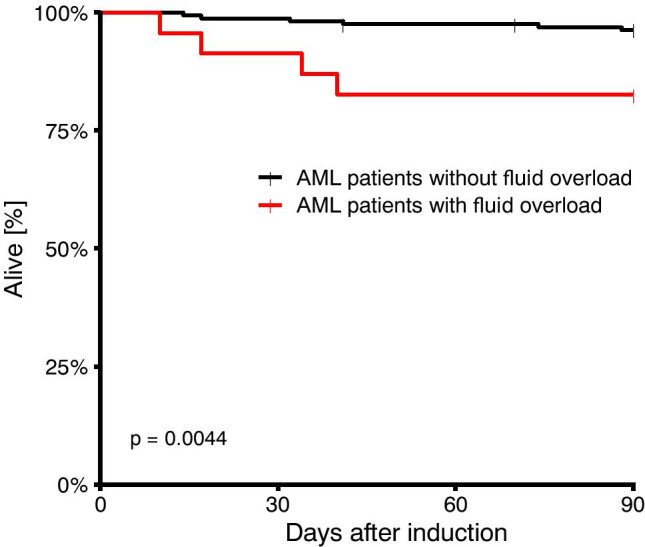
Kaplan-Meier estimates of 90-d overall survival (OS). The red line indicates OS in AML patients with FO; the black line indicates OS in AML patients without FO

AML patients with FO had a significantly poorer 90-day survival compared to patients without FO (*p*=0.0044) (Fig. 1). To further analyze risk factors for 90-day mortality in AML patients undergoing induction chemotherapy, a uni- and multivariate logistic regression model was performed. The nominal dichotome variables age above 60 years, treatment on ICU, adverse-risk AML, FO, patients receiving >100 ml crystalloid fluids/kg body weight, body weight >75 kg at day of admission, fever days >5, and CRP level >10 mg/dl were included in this model. As shown in Table 4, treatment on ICU and FO were independent risk factors for 90-day mortality in AML patients undergoing induction chemotherapy.

**Table 4:** Cox regression analysis of risk factors for 90-day mortality

Parameter	HR	95% CI	*P* value	HR	95% CI	*P* value
	Univariate analysis			Multivariate analysis		
**Age > 60 years**	1.543	0.454–5.245	0.487			
**Treatment on intensive care unit**	20.800	5.098–84.872	**<0.001**	33.144	6.147–178.713	**<0.001**
**Adverse-risk AML**	1.320	0.274–6.363	0.730			
**Fluid overload**	4.722	1.265–17.631	**0.021**	6.250	1.173–33.303	**0.032**
Patients receiving >100 ml crystalloid fluids/kg body weight*	1.961	0.554–6.938	0.296			
**Body weight > 75 kg at day of admission**	0.194	0.050–0.758	**0.018**			
**Fever days > 5 d**	1.000	0.294–3.399	1.000			
**CRP level > 10 mg/dl**	11.643	3.005–45.112	**0.003**			

*Including crystalloid fluids from day of admission until day 7 of induction chemotherapy. *CI*, confidence interval; *OR*, odds ratio. All *p*-values reported are two-sided. Statistical significance was defined as *p*≤0.05, significant p-values are presented in bold

Ten AML patients—4 (17.4%) AML patients with FO and 6 (3.7%) AML patients without FO—died within 90 days after induction chemotherapy. Causes for ICU transfer and death as well as the day 15 response status are shown in Table 5. Two (50%) of the 4 AML patients with FO died despite achieving bone marrow blast clearance on day 15 bone marrow evaluation; all 4 (100%) of them were transferred to ICU due to respiratory insufficiency. Respiratory insufficiency was due to pneumonia in 3 patients, and due to pulmonary edema in 1 patient. Three (75%) AML patients with FO died from acute respiratory distress syndrome (ARDS); 1 (25%) had hypoxic brain damage after resuscitation and epileptic seizures of unknown etiology. In AML patients without FO, 2 (33.3%) of 6 died despite achieving blast clearance on day 15 bone marrow evaluation. Two (33.3%) AML patients without FO died after the hospital stay for induction chemotherapy. The other 4 (66.7%) AML patients without FO were transferred to ICU. Two (50%) of these 4 patients were transferred to ICU due to respiratory insufficiency, and one due to toxic cardiomyopathy (probably due to anthracycline-containing induction chemotherapy) and the other one due to leukemic infiltration of the heart muscle, both finally leading to death. The other 2 AML patients without FO died from multi-organ failure after septic shock and from ARDS after aspiration pneumonia.

**Table 5:** Characterization of AML patients dying within 90 days after induction chemotherapy

**Patient**	**Fluid overload**	**Day 15 response**	**Reason for transfer to ICU**	**Cause of death during induction chemotherapy**
**1**	Yes	Yes	Respiratory insufficiency	ARDS
**2**	Yes	n/a	Respiratory insufficiency	ARDS and septic cardiomyopathy
**3**	Yes	Yes	Respiratory insufficiency	Hypoxic brain injury
**4**	Yes	No	Respiratory insufficiency	ARDS
**5**	No	No	Respiratory insufficiency	ARDS and toxic cardiomyopathy
**6**	No	No	Respiratory insufficiency	ARDS and cardiomyopathy
**7**	No	n/a	Respiratory insufficiency	ARDS and acute kidney injury
**8**	No	Yes	n/a	n/a
**9**	No	n/a	Septic shock	Multiple organ dysfunction syndrome
**10**	No	Yes	n/a	n/a

## Discussion

Numerous studies have demonstrated a strong correlation between FO and adverse outcomes like pulmonary edema, cardiac failure, impaired bowel function, and increased mortality in critically ill patients (25, 26). Although AML patients are prone to positive fluid balance, e.g., with the intention to prevent hyperuricemia-induced renal injury, tumor lysis syndrome (TLS), and leukostasis, to our knowledge, there is no study investigating the role of FO in AML patients undergoing induction chemotherapy. In this study, we identified increased administration of crystalloid fluids as a risk factor for FO on day 7 of induction chemotherapy and demonstrated that FO impairs survival in AML patients undergoing induction chemotherapy.

We defined FO as ≥ 5% weight gain on day 7 of induction chemotherapy with respect to a baseline body weight determined on the day of admission to hospital. Due to the retrospective nature of this study, considering clinical signs such as peripheral edema, high blood pressure, anasarca, or vena cava measurement to define FO was not feasible possibly. Since AML patients suffer from body weight loss during initial diagnosis and initiation of chemotherapy leading to possible underestimation of weight gain due to FO, we decided to choose a comparably low cutoff at 5% of body weight increase between admission and day 7 of induction chemotherapy. The reasoning behind screening for FO at the early timepoint on day 7 was to primarily detect FO caused by excess crystalloid fluids administered parallel to induction chemotherapy as this would be a potentially modifiable risk factor for FO. However, FO is multifactorial in etiology and is not necessarily a consequence of intravenous fluid administration. Amongst others, acute kidney injury, liver failure, congestive heart disease, or capillary leak due to inflammation or chemotherapy itself is clinical conditions known to lead to FO (27–30). Nevertheless, as induction chemotherapy is in general not initiated in a medical condition of severe organ dysfunction or uncontrolled sepsis, our screening timepoint intended to focus on FO in the context of unthoughtful fluid administration due to obedience to protocol recommendation. Notably, current AML treatment protocols recommend to regularly apply 1000 ml/day of crystalloid fluids as concomitant medication during induction chemotherapy (17).

In total, we found FO in 23 (12%) of 187 AML patients undergoing induction chemotherapy. Meaningful comparisons of FO incidence to other hematological studies investigating FO are difficult. Chamoun et al., Miller et al., and Rondon et al. detected a FO incidence of 14%, 28.6%, and 66.2% in essentially different patient cohorts, with different screenings and definitions for FO (12–14). As mentioned above, the FO definition in this study (solely based on weight gain) and its screening timepoint (limited to day 7 of induction chemotherapy) as well as the assumption that AML patients suffer from weight loss during the screening episode (due to active cancer disease, chemotherapy-induced nausea and vomiting, etc.) lead to potential underestimation of FO incidence.

AML patients with FO received in median 1000 ml more of crystalloid fluids until day 7 of induction chemotherapy than AML patients without FO. This difference was not statistically significant. However, when put in relation to body weight, AML patients with FO received more often over 100 ml of crystalloid fluids per kg body weight until day 7 of induction chemotherapy, which was identified as an independent risk factor for FO. Considering that the fraction of AML patients with FO presenting with a body weight < 75 kg was significantly higher than that of AML patients without FO, we conclude that more thoughtful and weight-adjusted prescription of crystalloid fluids is an easy and obvious way to reduce risk for FO in daily clinical practice. Importantly, AML patients with FO suffered from FO despite receiving significantly more diuretics than AML patients without FO.

As prophylactic and therapeutic administration of RBC and platelet concentrates constitute a cornerstone of supportive measures in AML patients, we analyzed the median blood transfusions administered until day 7 of induction chemotherapy in AML patients with and without FO. The median transfusion rates for platelet and RBC concentrates were twice as high in AML patients with FO as in AML patients without FO. It remains unclear whether the higher blood transfusion rates are an additional cause for FO in AML patients with FO or if the higher transfusion rate actually represents avoidable blood transfusions deemed indicated by numeric thresholds that were reached more frequently due to hemodilution (31). Randomized prospective studies will be needed to analyze and understand this interesting observation in more detail.

CRP levels at the day of admission did not differ significantly between AML patients with and without FO, although there was a trend towards higher CRP levels in AML patients with FO (*p*=0.084). This trend possibly demonstrates that AML patients with FO had more inflammation (e.g., due to infection or active leukemia) from the beginning potentially tempting the clinician in charge to prescribe more crystalloid fluids. When considering median CRP levels of the total hospital stay for induction chemotherapy, AML patients with FO had twice as high CRP levels as AML patients without FO. In critically ill patients, FO is described as a risk factor for the development of inflammation. The concomitance of FO and inflammation was correlated with an increased risk of death. Accordingly, elevated CRP and Il-6 levels were described in critically ill patients with FO (32, 33). Due to the retrospective nature of this study, it cannot be finally determined whether AML patients with a higher CRP need more crystalloid fluids and FO is a largely unavoidable consequence or whether FO promotes infection and inflammation reflected by significantly higher CRP levels. Prospective studies are urgently needed to unravel the net relationship between elevated CRP levels and FO in order to wisely adapt fluid management in AML patients with inflammation.

Treatment on ICU and FO were independent risk factors for 90-day mortality in AML patients undergoing induction chemotherapy, which accentuates the role of FO as a modifiable risk factor for treatment outcome. Within the 10 AML patients—4 AML patients with FO and 6 AML patients without FO—dying within 90 days after induction chemotherapy, we found that AML patients with FO were likely to die despite the absence of uncontrolled leukemia disease and are susceptible to respiratory insufficiency and pneumonia leading to ICU treatment (Table 5). There are several limitations to this analysis. Firstly, the number of AML patients dying within 90 days after AML induction chemotherapy is fortunately very small. Secondly, as AML patients on ICU mostly suffer from sepsis, multiple organ dysfunction, and/or uncontrolled leukemia disease, unambiguously determining the leading cause for death in these patients is hardly possible in a retrospective study setting. Comparing these observations with respect to the presence or absence of FO is therefore not appropriate. The association between FO and an increased early mortality has been described in critically ill patients (34–36). On the one hand, the administration of adequate fluid resuscitation might help prevent acute kidney injury due to limited renal perfusion and might be essential to achieve restoration of cardiac output and an adequate systemic blood pressure in patients with cardiogenic or septic shock (37). On the other hand, critically ill patients are prone to increased capillary leak secondary to inflammation (release of cytokines, complement factors, and altered organ microcirculation). This leads to administered intravenous fluid leaving the circulatory system and causing edema, which has been shown to result in impaired oxygen and metabolite diffusion and consequently in progressive organ dysfunction (38, 39).

AML patients are susceptible to hyperhydration due to crystalloid fluids applied in the context of chemotherapy protocols, during sepsis treatment, or due to blood transfusions. Traditionally, AML patients receive crystalloid fluids to prevent TLS. However, Miller et al. have illustrated that a significant proportion of pediatric patients received hyperhydration although they were not at high risk for developing TLS (13). It is important to note that conservative fluid balance strategies have been successfully applied in critically ill patients, including sepsis patients, stem cell transplant recipients, and trauma patients. Therefore, it may be well-founded to reassess widely implemented practices on fluid management also for AML patients undergoing induction chemotherapy (8, 40–42). Due to the adverse impact of FO which is a modifiable risk factor on the clinical course of AML patients undergoing induction therapy, we advocate that hematologists should be more vigilant for the signs and symptoms of FO, and carefully monitor body weight gain. We suggest an individualized, hemodynamically guided, and restrictive approach to fluid administration to reduce preventable treatment-related morbidity and mortality. The prevention of AML-related complications such as leukostasis and TLS is essential; however, we suggest a strict stratification of patients requiring hyperhydration. We further advise the accurate documentation of increase in weight in relation to the body weight and the early identification of patients who are susceptible to FO.

As to our limitations, the retrospective nature of our study limits the conclusions that can be drawn from the exhibited results. Clinical signs such as peripheral edema, high blood pressure, anasarca, or vena cava measurement and especially oral fluid intake were not considered for our definition of FO as they were not documented in a standardized manner for all AML patients. Prospective studies are needed to determine the precise incidence of FO in AML patients and to analyze whether the recommended strategies will help decrease the incidence of FO in AML patients undergoing induction chemotherapy.

## References

[1] Abrahão R, Keogh RH, Lichtensztajn DY, Marcos-Gragera R, Medeiros BC, Coleman MP (2016). Predictors of early death and survival among children, adolescents and young adults with acute myeloid leukaemia in California, 1988–2011: a population-based study. Br J Haematol..

[2] Bradstock KF, Matthews JP, Lowenthal RM, Baxter H, Catalano J, Brighton T (2005). A randomized trial of high-versus conventional-dose cytarabine in consolidation chemotherapy for adult de novo acute myeloid leukemia in first remission after induction therapy containing high-dose cytarabine. Blood..

[3] Pemmaraju N, Kantarjian H, Garcia-Manero G, Pierce S, Cardenas-Turanzas M, Cortes J (2015). Improving outcomes for patients with acute myeloid leukemia in first relapse: a single center experience. Am J Hematol..

[4] Sivendran S, Latif A, McBride RB, Stensland KD, Wisnivesky J, Haines L (2014). Adverse event reporting in cancer clinical trial publications. J Clin Oncol..

[5] Buckley SA, Othus M, Estey EH, Walter RB (2015). The treatment-related mortality score is associated with non-fatal adverse events following intensive AML induction chemotherapy. Blood Cancer J.

[6] Othus M, Kantarjian H, Petersdorf S, Ravandi F, Godwin J, Cortes J (2014). Declining rates of treatment-related mortality in patients with newly diagnosed AML given ‘intense’ induction regimens: a report from SWOG and MD Anderson. Leukemia..

[7] Boyd JH, Forbes J, Nakada TA, Walley KR, Russell JA (2011). Fluid resuscitation in septic shock: a positive fluid balance and elevated central venous pressure are associated with increased mortality. Crit Care Med..

[8] Cordemans C, De Laet I, Van Regenmortel N, Schoonheydt K, Dits H, Huber W (2012). Fluid management in critically ill patients: the role of extravascular lung water, abdominal hypertension, capillary leak, and fluid balance. Ann Intensive Care..

[9] Díaz F, Nuñez MJ, Pino P, Erranz B, Cruces P (2018). Implementation of preemptive fluid strategy as a bundle to prevent fluid overload in children with acute respiratory distress syndrome and sepsis. BMC Pediatr..

[10] Ávila MO, Rocha PN, Zanetta DM, Yu L, Burdmann Ede A (2014). Water balance, acute kidney injury and mortality of intensive care unit patients. J Bras Nefrol..

[11] Stucki A, Rivier AS, Gikic M, Monai N, Schapira M, Spertini O (2001). Endothelial cell activation by myeloblasts: molecular mechanisms of leukostasis and leukemic cell dissemination. Blood..

[12] Chamoun K, Kantarjian HM, Wang X, Naqvi K, Aung F, Garcia-Manero G (2019). Unrecognized fluid overload during induction therapy increases morbidity in patients with acute promyelocytic leukemia. Cancer..

[13] Miller LH, Keller F, Mertens A, Klein M, Allen K, Castellino S (2019). Impact of fluid overload and infection on respiratory adverse event development during induction therapy for childhood acute myeloid leukemia. Pediatr Blood Cancer.

[14] Rondon G, Saliba RM, Chen J, Ledesma C, Alousi AM, Oran B (2017). Impact of fluid overload as new toxicity category on hematopoietic stem cell transplantation outcomes. Biol Blood Marrow Transplant..

[15] Rondon-Clavo C, Scordo M, Hilden P, Shah GL, Cho C, Maloy MA (2018). Early fluid overload is associated with an increased risk of nonrelapse mortality after ex vivo CD34-selected allogeneic hematopoietic cell transplantation. Biol Blood Marrow Transplant..

[16] Wiernik PH, Case DC, Periman PO, Arlin ZA, Weitberg AB, Ritch PS (1989). A multicenter trial of cytarabine plus idarubicin or daunorubicin as induction therapy for adult nonlymphocytic leukemia. Semin Oncol..

[17] Engelhardt M, Berger DP, Mertelsmann R, Duyster J (eds) Das Blaue Buch, edn 6. Springer, Berlin, Heidelberg. 10.1007/978-3-662-51420-7

[18] Hiddemann W, Kreutzmann H, Straif K, Ludwig WD, Mertelsmann R, Donhuijsen-Ant R (1987). High-dose cytosine arabinoside and mitoxantrone: a highly effective regimen in refractory acute myeloid leukemia. Blood..

[19] Krug U, Buchner T, Berdel WE, Muller-Tidow C (2011). The treatment of elderly patients with acute myeloid leukemia. Dtsch Arztebl Int..

[20] Dohner H, Estey EH, Amadori S, Appelbaum FR, Buchner T, Burnett AK (2010). Diagnosis and management of acute myeloid leukemia in adults: recommendations from an international expert panel, on behalf of the European LeukemiaNet. Blood..

[21] Neumann S, Krause SW, Maschmeyer G, Schiel X, von Lilienfeld-Toal M, Infectious Diseases Working P (2013). Ann Hematol.

[22] Mellinghoff SC, Panse J, Alakel N, Behre G, Buchheidt D, Christopeit M (2018). Primary prophylaxis of invasive fungal infections in patients with haematological malignancies: 2017 update of the recommendations of the Infectious Diseases Working Party (AGIHO) of the German Society for Haematology and Medical Oncology (DGHO). Ann Hematol..

[23] Freifeld AG, Bow EJ, Sepkowitz KA, Boeckh MJ, Ito JI, Mullen CA (2011). Clinical practice guideline for the use of antimicrobial agents in neutropenic patients with cancer: 2010 update by the infectious diseases society of america. Clin Infect Dis..

[24] Ballo O, Fleckenstein P, Eladly F, Kreisel EM, Stratmann J, Seifried E (2020). Reducing the red blood cell transfusion threshold from 8.0 g/dl to 7.0 g/dl in acute myeloid leukaemia patients undergoing induction chemotherapy reduces transfusion rates without adversely affecting patient outcome. Vox Sang.

[25] Claure-Del Granado R, Mehta RL (2016). Fluid overload in the ICU: evaluation and management. BMC Nephrol..

[26] Vaara ST, Korhonen AM, Kaukonen KM, Nisula S, Inkinen O, Hoppu S (2012). Fluid overload is associated with an increased risk for 90-day mortality in critically ill patients with renal replacement therapy: data from the prospective FINNAKI study. Crit Care..

[27] Ostermann M, Straaten HM, Forni LG (2015). Fluid overload and acute kidney injury: cause or consequence?. Crit Care..

[28] Rzouq F, Alahdab F, Olyaee M (2014). New insight into volume overload and hepatorenal syndrome in cirrhosis, “the hepatorenal reflex hypothesis”. Am J Med Sci..

[29] Pellicori P, Kaur K, Clark AL (2015). Fluid management in patients with chronic heart failure. Card Fail Rev..

[30] Wu MA, Colombo R, Podda GM, Cicardi M (2019). Handling shock in idiopathic systemic capillary leak syndrome (Clarkson’s disease): less is more. Intern Emerg Med..

[31] Perel A (2017). Iatrogenic hemodilution: a possible cause for avoidable blood transfusions?. Crit Care..

[32] Dekker MJE, van der Sande FM, van den Berghe F, Leunissen KML, Kooman JP (2018). Fluid overload and inflammation axis. Blood Purif..

[33] Demirci MS, Demirci C, Ozdogan O, Kircelli F, Akcicek F, Basci A (2011). Relations between malnutrition-inflammation-atherosclerosis and volume status. The usefulness of bioimpedance analysis in peritoneal dialysis patients. Nephrol Dial Transplant.

[34] Goldstein SL, Somers MJ, Baum MA, Symons JM, Brophy PD, Blowey D (2005). Pediatric patients with multi-organ dysfunction syndrome receiving continuous renal replacement therapy. Kidney Int..

[35] Gomes J, Pesavento ML, de Freitas FFM, de Andrade Coelho FU (2019). Fluid overload and risk of mortality in critically ill patients. Dimens Crit Care Nurs.

[36] Messmer AS, Zingg C, Müller M, Gerber JL, Schefold JC, Pfortmueller CA (2020). Fluid overload and mortality in adult critical care patients-a systematic review and meta-analysis of observational studies. Crit Care Med..

[37] Prowle JR, Echeverri JE, Ligabo EV, Ronco C, Bellomo R (2010). Fluid balance and acute kidney injury. Nat Rev Nephrol..

[38] Andreucci M, Federico S, Andreucci VE (2001). Edema and acute renal failure. Semin Nephrol..

[39] Boyle A, Maurer MS, Sobotka PA (2007). Myocellular and interstitial edema and circulating volume expansion as a cause of morbidity and mortality in heart failure. J Card Fail..

[40] Michael M, Kuehnle I, Goldstein SL (2004). Fluid overload and acute renal failure in pediatric stem cell transplant patients. Pediatr Nephrol..

[41] Murphy CV, Schramm GE, Doherty JA, Reichley RM, Gajic O, Afessa B (2009). The importance of fluid management in acute lung injury secondary to septic shock. Chest..

[42] Silversides JA, Major E, Ferguson AJ, Mann EE, McAuley DF, Marshall JC (2017). Conservative fluid management or deresuscitation for patients with sepsis or acute respiratory distress syndrome following the resuscitation phase of critical illness: a systematic review and meta-analysis. Intensive Care Med..

